# BEYOND THE STARTING LINE: AN INTERSECTIONAL LIFE COURSE ANALYSIS OF OLDER WOMEN RECREATIONAL RUNNERS IN RURAL MAINE

**DOI:** 10.1093/geroni/igae098.4132

**Published:** 2024-12-31

**Authors:** Jennifer Jain

**Affiliations:** University of Maine, Orono, Maine, United States

## Abstract

This study offers a close examination of the experiences of women runners, age 65 and older, who have witnessed the vast changes to the landscape of recreation and sports across their lifespans. The enactment of Title IX in 1972 made it more acceptable for women to pursue an active lifestyle. This qualitative study uses a grounded theory approach to examine the experiences of 13 women runners, ages 65-78, who live in rural Maine. The study aimed to understand the turning points along their life course that inspired them to run, the personal and social challenges, and the strategies that enabled them to continue running. The women participated in focus groups, journaled, and participated in follow-up interviews. Preliminary findings show that many older women were introduced to unorganized sports through family members at a young age. As adults, they turned to recreational running to maintain and improve their mental and physical health. Older women runners face challenges such as health issues, concerns related to public perception of aging bodies, slowed running pace that impedes participation in club running, and a lack of running companions due to living in rural locations. Despite their challenges, a majority of the women continued to run by drawing upon their commitment, planning, and preparations, self-awareness of their body’s abilities, and acceptance and adaptation to challenges. The results of this study can help to improve future community programming and lower the systemic and geographical barriers to maintaining physical activity for older women.

